# Comprehensive Risk Assessment of Typical High-Temperature Cities in Various Provinces in China

**DOI:** 10.3390/ijerph19074292

**Published:** 2022-04-03

**Authors:** Xueru Zhang, Qiuyue Long, Dong Kun, Dazhi Yang, Liu Lei

**Affiliations:** 1 School of Public Administration, Hebei University of Economics and Business, Shijiazhuang 050061, China; dongkun1113@heuet.edu.cn (D.K.); hbsjzliul@gmail.com (L.L.); 2 Collaborative Innovation Center of Urban-Rural Integration Development, Hebei University of Economics and Business, Shijiazhuang 050061, China; 3 Chongqing Cybercity Sci-Tech Co., Ltd., Chongqing 401121, China; longqy0815@gmail.com; 4 Key Laboratory of Land Surface Pattern and Simulation, Institute of Geographic Sciences and Natural Resources Research, Chinese Academy of Sciences, Beijing 100101, China; yangdazhi@igsnrr.ac.cn; 5 College of Resources and Environment, University of Chinese Academy of Sciences, Beijing 100049, China

**Keywords:** GIS, high-temperature disaster risk, high-temperature disaster vulnerability, risk assessment

## Abstract

Global climate change results in an increased risk of high urban temperatures, making it crucial to conduct a comprehensive assessment of the high-temperature risk of urban areas. Based on the data of 194 meteorological stations in China from 1986 to 2015 and statistical yearbooks and statistical bulletins from 2015, we used GIS technology and mathematical statistics to evaluate high-temperature spatial and temporal characteristics, high-temperature risk, and high-temperature vulnerability of 31 cities across China. Over the past 30 years, most Chinese cities experienced 5–8 significant oscillation cycles of high-temperature days. A 15-year interval analysis of high-temperature characteristics found that 87% of the cities had an average of 5.44 more high-temperature days in the 15-year period from 2001 to 2015 compared to the period from 1986 to 2000. We developed five high-temperature risk levels and six vulnerability levels. Against the background of a warming climate, we discuss risk mitigation strategies and the importance of early warning systems.

## 1. Introduction

High-temperature disaster refers to a meteorological disaster that causes discomfort to living and non-living things such as people, animals, plants, and inorganic environment due to extreme high-temperature weather and has adverse effects. Global climate change has resulted in more frequent extreme weather events, which have significant adverse effects on human health and the social economy [1]. One type of extreme climate event, extreme summer heat, is a global phenomenon: In 1993, the Southeastern United States was hit by heat waves, with most areas reaching their highest temperatures in history [2]. In 1994, continuous high summer temperatures in Northeast China, Japan, and other countries resulted in large-scale droughts [3]. In 1995, the heat wave across Europe seriously impacted ecological, socio-economic, and other aspects of society [4]. In the summer of 2003, temperatures in Europe hit a record high [5], with more than 10,000 people in France dying as a consequence of the heat [6]. In 2013, Southeast China suffered from abnormally high temperatures, and 167 excess deaths occurred in the Pudong New Area in Shanghai [7]; at the same time, 679 additional heat-related illnesses occurred in Ningbo [8]. Heat waves, which are occurring more frequently, affect not only human health [9,10] but also human wellbeing and productivity, resulting in urban water and power supply shortages [11] and, consequently, threatening food security [12]. Over the last few years, high-temperature events have become more frequent, and their frequency, range, and duration will continue to increase [13]. Analyzing the spatial and temporal characteristics of high-temperature events can facilitate our understanding of the intensity, frequency, and duration of such events and their causes. In this sense, high-temperature risk assessment is of great importance for avoiding the risks associated with high-temperature events.

Studies on high-temperature events mainly focused on the impacts and causes of these events, as well as early warning systems. For example, Rosenzweig et al. [13,14] pointed out that urban heat waves can have serious impacts on human health. According to Park et al. [15] and Guan et al. [3], the occurrence of high-temperature events is related to abnormal sea temperatures. While the US Hot-Weather Health Warning System (HHWS) can already assess the possible number of deaths caused by hot weather [16], Kalkstein et al. [17] evaluated the ability of HHWS to reduce the number of heat-related deaths based on the number of heat deaths and heat waves in major cities in the United States from 1975 to 2004. Yang et al. [18] pointed out that the urban heat island effect aggravated the scope and intensity of extremely high temperatures in cities, increased high-temperature health risks for urban residents, and also made an important contribution to the long-term upward trend of extremely high temperatures in cities.

High-temperature vulnerability research refers to the establishment of a vulnerability evaluation index system in terms of the sensitivity; exposure; and adaptability of natural resources, the environment, the population, and the social economy, with the aim of quantitatively expressing the regional high-temperature vulnerability and identifying its spatial distribution [19,20]. Against the background of global climate change, compared to vulnerability assessments of meteorological disasters such as floods, droughts, and typhoons, as well as geological disasters such as earthquakes and landslides, studies assessing the vulnerability of cities to high-temperature events are scarce [21,22,23,24,25,26,27,28]. Urban high-temperature vulnerability evaluation studies mainly used official statistics, obtained via remote sensing and GIS. Generally, indicators such as high-temperature stress, sensitivity, adaptability, and exposure were evaluated [29,30], and factors such as the number of high-temperature days, socioeconomic level, and education level were involved [19,20]. The determination of relevant evaluation indicators is highly subjective, requiring rigorous index demonstration, mostly using statistical data; remote sensing data and GIS data alone are insufficient [31].

Disaster risk assessment is a process of judging the nature and scope of risk by studying the disaster-causing factors and the vulnerability of disaster-bearing bodies that have potential impacts on life, property and environment [32]. Considering the combined effects of risk and vulnerability, the comprehensive risk of high temperature disaster pays more attention to the possible losses under high temperature stress; that is, it emphasizes the exposure of population, property, and ecosystem at high temperatures [20]. In the 1970s, several countries started to conduct risk assessments of meteorological disasters [33,34,35,36,37,38]. For example, Blaikei et al. [39] took each state as the research object and used natural disasters between 1957 and 1994 to conduct a natural disaster risk analysis, using disaster loss, population, and area data. The relationship between resource development and natural disasters was illustrated from the perspective of the comprehensive role of the disaster-prone environment, disaster-causing factors, and disaster-bearing factors, allowing the authors to obtain the disaster risk zoning of the United States. In 1982, Willam et al. [40] completed the book *Natural Disaster Risk Assessment and Disaster Reduction Policy,* in which the authors described natural disaster risk assessment. China’s high-temperature risk assessment has mainly been carried out by considering hazards, exposure, vulnerability, disaster prevention, mitigation ability, etc., and high-temperature risk assessment is performed by considering disaster-causing factors, disaster-prone environments, disaster-bearing bodies, disaster resistance ability, etc. To date, although several studies have investigated the influences and causes of high-temperature events, including potential early warning signs [13,14,15,16,17], systematic studies on the risk assessment of high-temperature events are scarce.

The above-mentioned studies mainly focused on spatial-temporal characteristics, causes, impacts on human health, and a comprehensive assessment of high-temperature events. However, such research was largely carried out on the regional scale. On the national scale, based on different high-temperature risk assessment models and different evaluation index systems, this paper constructs a high-temperature risk assessment model from the comprehensive perspective of high-temperature spatiotemporal characteristics, risks, and vulnerability. We used statistical methods to analyze high-temperature spatiotemporal characteristics and risk assessment data from various cities within China based on provincial units. The identification of areas vulnerable to high temperatures and the assess of this vulnerability provide a scientific basis for the control of high-temperature risks in various cities with a high practical significance.

## 2. Data and Methods

### 2.1. Data

**Figure 1:**
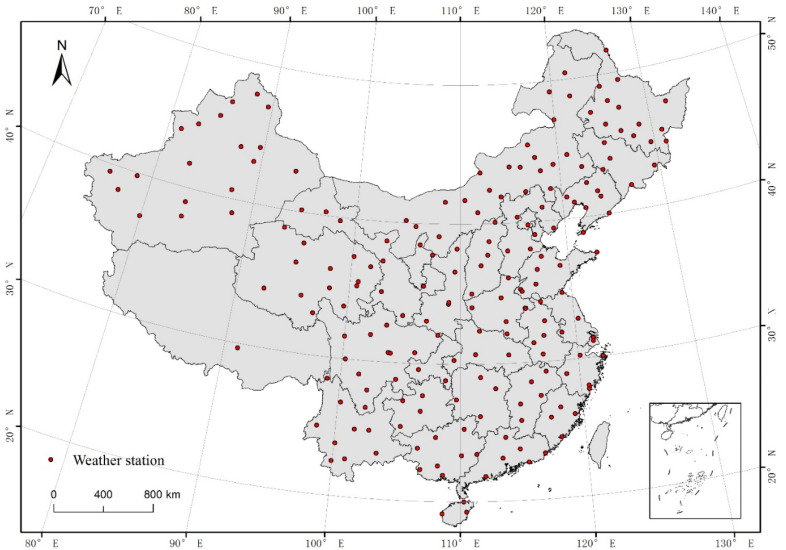
Map showing the spatial distribution of weather stations across China.

For this paper, we used meteorological and socioeconomic data. Meteorological data were obtained from the National Meteorological Science Data Sharing Center (http://data.cma.cn/site/index.html, accessed on 10 April 2020); we downloaded the daily maximum temperature data for 194 weather stations from the “China Ground International Exchange Station Climate Data Day Dataset” from 1986 to 2015 (Figure 1). Social and economic data were obtained from the statistical yearbooks of various provinces and cities, supplemented by departmental statistical yearbooks and statistical bulletins (Table 1).

### 2.2. Methods

The fifth research report of the IPCC (2014) emphasizes the importance of risk assessment in global climate change research, describing a framework of natural disaster risk assessment based on “disaster stress-social vulnerability-exposure.” Extremely high temperatures in summer can lead to high-temperature disasters, which pose serious threats to human health, the social economy, and ecosystems. Based on the natural disaster risk evaluation framework and “high-temperature risk-social vulnerability-population exposure,” a high-temperature disaster risk assessment framework is constructed to comprehensively assess high-temperature risks. According to the above evaluation framework, the quantitative analysis of high-temperature characteristics, risks, and vulnerability is conducted to provide an assessment of urban high-temperature risks.

#### 2.2.1. Analysis of High-Temperature Characteristics

Based on the daily maximum temperature data of 194 weather stations from the “China Ground International Exchange Station Climate Data Dataset” from 1986 to 2015, 31 cities were selected as typical cities with high temperatures, and the frequency characteristics of high-temperature events in these cities were determined. We used the SPSS software platform for statistical analysis and counted the days with high-temperature events from 1986 to 2015. In order to observe and compare these events, we used the equidistant grouping method that is frequently adopted [41]. The obtained dataset was divided into two groups, namely data from 1986 to 2000 and data from 2001 to 2015. For each group, which represents a 15-year period, we calculated the mean of the annual number of high-temperature days. Subsequently, we calculated the difference between the average number of high-temperature days from 2001 to 2015 and the average number of high-temperature days from 1986 to 2000 and compared the two 15-year periods.

#### 2.2.2. Risk Analysis of High-Temperature Disasters

Using the temperature data of 31 typical stations, a high-temperature risk assessment model for 31 typical provinces and cities was constructed based on the following three aspects: the duration of high temperatures, high-temperature severity, and extreme high-temperature risk. The duration of high-temperature events was expressed by the number of high-temperature days (≥35 °C). The severity of a high-temperature event is expressed by the average difference between the daily maximum temperature (when it is ≥35 °C) and the temperature of 35 °C. The extreme high-temperature risk was expressed by the extreme high temperature ratio, which refers to the ratio of the number of days with a maximum daily temperature of ≥38 °C (China’s Meteorological Administration defines weather with a daily maximum temperature ≥38 °C as hot summer weather) to the number of high-temperature days within a certain period of time. The model is constructed as follows:

*R* = *d* × *w*_*d*_ + *t* × *w*_*t*_ + *p* × *w*_*p*_ (1)

where *R* indicates the risk of urban high-temperature disasters; and *d*, *t*, and *p* represent the cumulative number of high-temperature days, the high-temperature severity, and the extreme high-temperature ratio of the standardized cities, respectively. *w_d_*, *w_t_*, and *w_p_* are the standardized weights of the cumulative number of high-temperature days, high-temperature severity, and extreme high-temperature ratio for each city, respectively. The cumulative number of high-temperature days is the number of days with high-temperature weather; the high-temperature severity is the average of the difference between the daily maximum temperature (when it is ≥35 °C) and 35 °C; and the extreme high-temperature ratio refers to the ratio of the number of days with a daily maximum temperature ≥38 °C to the number of high-temperature days in a certain period of time.

In a previous study [19], *w_d_*, *w_t_*, and *w_p_* were set to 0.6, 0.3, and 0.1, respectively. Based on the calculated risk index values of each typical city, the natural breakpoint method in ArcGIS was used to grade cities, and the spatial distribution maps of five high-temperature risk grades were obtained.

#### 2.2.3. Vulnerability Analysis of High-Temperature Disaster and High-Temperature Risk Assessment

According to the high-temperature risk assessment framework, the risk can be determined using the following three indicators: high-temperature stress, social vulnerability, and population exposure. Social vulnerability refers to how vulnerable a specific group of people is to high temperatures and their ability to resist high-temperature hazards; it includes the sensitivity and adaptability of the population [42]. Based on previous studies, the multiplication and division of these indicators can reflect the synergistic relationships among the indicators more effectively than addition and subtraction [43,44,45,46]. Accordingly, the high-temperature vulnerability and the high-temperature risk models were obtained as follows:

*DI* = *R* × *F*, and (2)

*RI* = *R* × *F* × *E*, (3)

where *DI* is the high-temperature vulnerability index; *RI* is the high-temperature risk index; and *R*, *F*, and *E* are the high-temperature disaster risk, social vulnerability, and population exposure values, respectively. Parameter R is calculated using Equation (1); population exposure value E refers to the total population of each city in 2015. Social vulnerability, F, is determined via principal component analysis, which is used to analyze and calculate the selected social vulnerability indicators of sensitivity and adaptability; the specific indicators are shown in Appendix A. Principal component analysis (PCA) [47] is a multivariate dimensionality reduction technique for clustering and index reduction that uses the relationships between data points; it simplifies and organizes the relationships among a set of metrics and, thus, enables objective index confirmation. To calculate social vulnerability, the principal component score function of each typical site was first calculated based on the resulting component score coefficient matrix:

*F_i_* = ∑ *Z_ij_* × *X* (4)

where *X* indicates the value of each index after standardization and *Z_ij_* is the corresponding component score of the index.

Subsequently, we used the contribution rates of each principal component and calculated the social vulnerability value *F* of each city by applying the following equation:

(5) $$F=\frac{e_{i}}{e}F_{i}$$

where *e_i_* is the contribution rate of each principal component; *e* represents the total principal component contribution rate; and *F_i_* represents each principal component score.

Based on the constructed high-temperature risk assessment framework, the high-temperature vulnerability and high-temperature risk values of the cities with the highest temperatures in various provinces were obtained. The natural breakpoint method in ArcGIS was used to generate the maps of high-temperature vulnerability and high-temperature risk zoning for each city (Table 2).

#### 2.2.4. Jenks Natural Breaks

The natural breakpoint method is a standard method used to divide datasets into a certain number of classes; it is widely used in data analysis and map making [48]. By identifying the classification interval and dividing the elements into multiple classes, similar values can be appropriately grouped so that the difference between similar groups are small and the differences between less similar groups are large. Statistically, the variance can be used to perform the classification. The magnitude of the sum of the variance of various classifications can be used to classify the elements, and the lowest magnitude indicates the best classification result. The natural breaks method is the “best” method for finding an appropriate segmentation range. Most high-temperature risk studies divide the risk into five levels [47,49], which are not arbitrary decisions. Therefore, in this paper, five high-temperature risk levels are determined, and the thresholds of all levels are obtained using the natural breaks method.

## 3. Results

### 3.1. Analysis of the Spatial and Temporal Characteristics of Urban High-Temperature Events

**Figure 2:**
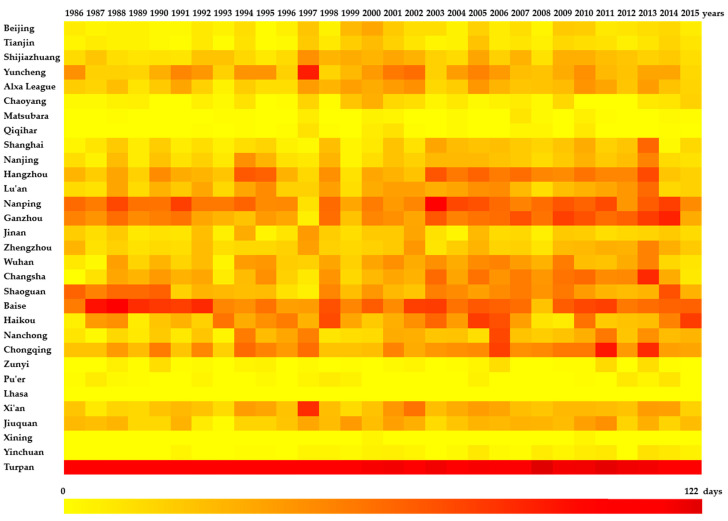
Number of high-temperature days (≥35 °C) in 31 typical Chinese cities from 1986 to 2015.

**Figure 3:**
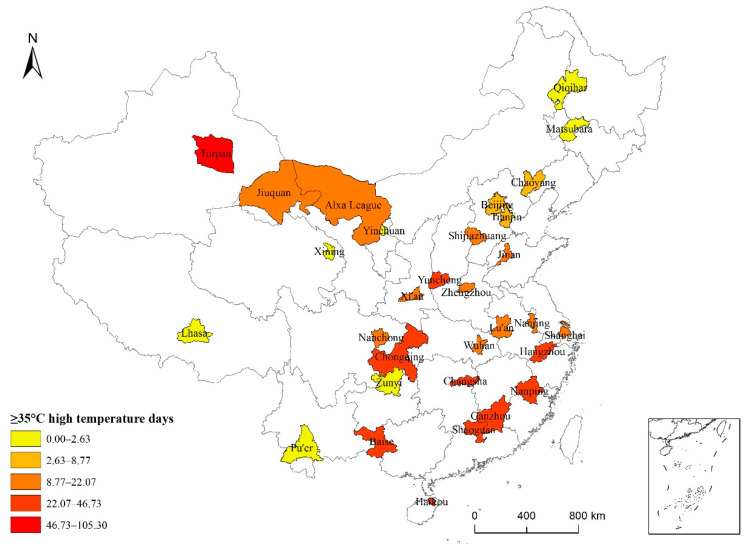
Spatial characteristics of high-temperature days in 31 typical cities in China from 1986 to 2015.

Generally, 28 of the 31 typical cities showed cyclical fluctuations in the number of high-temperature days per year (Figure 2 and Figure 3), and most cities experienced 5–8 significant oscillation cycles. Turpan, Xinjiang, had the largest number of high-temperature days per year, with a mean of 105.30 days. The number of high-temperature days per year in Baise, Guangxi; Nanping, Fujian; Ganzhou, Jiangxi; Chongqing; and Hangzhou, Zhejiang, showed obvious cyclical fluctuations, with a mean of greater than 30 days. Turpan had an average of 30.03 to 46.73 high-temperature days. Shaoguan, Guangdong; Haikou, Hainan; Changsha, Hunan; Yuncheng, Shanxi; Wuhan, Hubei; Lu’an, Anhui; and Xi’an, Shaanxi, had 22 to 28.8 high-temperature days per year on average. Alxa League in Inner Mongolia; Nanchong, Sichuan; Jiuquan, Gansu; Zhengzhou, Henan; Shijiazhuang, Hebei; Nanjing, Jiangsu; Shanghai, Shandong; and Jinan, Shandong, averaged between 10 and 20 high-temperature days per year. Beijing; Tianjin; Chaoyang, Liaoning; Yinchuan, Ningxia; Zunyi, Guizhou; Pu ’er, Yunnan; Qiqihar, Heilongjiang; and Songyuan, Jilin, averaged between 1 and 10 high-temperature days per year. In every year, the number of high-temperature days was below 40 for these cities.

According to the number of high-temperature days, natural breakpoints were used in ArcGIS to create five intensity levels: very low, low, medium, high, and very high. From a spatial perspective, Turpan in Northwest China is a very high-intensity area. Most cities in South China, East China, and the Yangtze River basin are high-intensity areas, whereas cities in North China and Northeast China are medium- and low-intensity areas.

**Figure 4:**
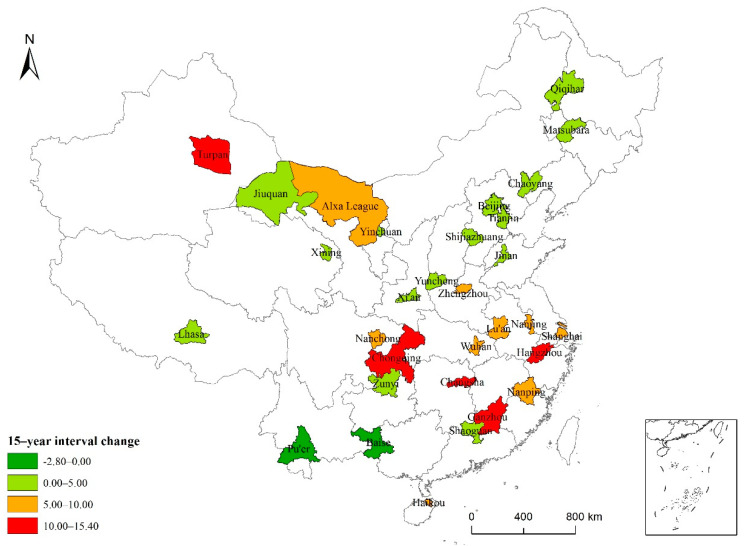
Map of China showing the 15-year interval variation of high-temperature weather in typical cities.

The results of the time series analysis of high-temperature events in the 31 typical cities are shown in Table 3. For Tianjin, Shanghai, Ganzhou, Zhengzhou, Changsha, Nanchong, Chongqing, Yinchuan, and Turpan, the number of high-temperature days gradually increased between 1986 and 2015. However, for 87.10% of the cities, we found an increase in the average number of high-temperature days from 2001 to 2015 compared to 1986–2000 (Figure 4); in two cities, the number of high-temperature days per year decreased. In Changsha, Ganzhou, Chongqing, Hangzhou, and Turpan, the number of high-temperature days increased significantly by more than 10 days; in Shanghai, Wuhan, Nanchong, Nanping, Lu’an, Haikou, Zhengzhou, Alxa League, and Nanjing, this number increased by 5.13–9.67 days. In contrast, in Beijing, Tianjin, Shijiazhuang, Yuncheng, Chaoyang, Songyuan, Qiqihar, Jinan, Shaoxing, Zunyi, Lhasa, Xi’an, Jiuquan, and Yinchuan, a significant increase of 0–5 days was found. The average number of high-temperature days in Lhasa and Xining was the same for the two 15-year periods. In Baise and Pu’er, the number of high-temperature days decreased by 2.80 and 0.33 days, respectively. The cities with a large increase in the number of high-temperature days were mainly located in East China, South China, and the Yangtze River basin.

### 3.2. Risk and Vulnerability Analysis

**Figure 5:**
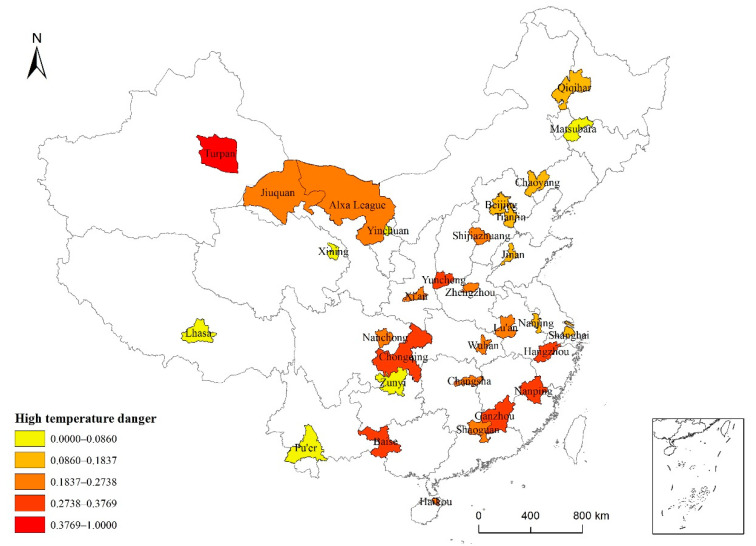
High-temperature risk levels for 31 typical cities across China based on data from 1986 to 2015.

Using ArcGIS combined with the obtained risk index value R, the natural breakpoint method was applied to divide the risk into five levels from high to low (Figure 5) as follows: Turpan—Level V (very high); Yuncheng, Hangzhou, Nanping, Ganzhou, Chongqing, and Baise—Level IV (high); Shijiazhuang, Alxa League, Lu’an, Zhengzhou, Wuhan, Changsha, Shaoguan, Haikou, Nanchong, Xi’an, and Jiuquan—Level III (medium); Beijing, Tianjin, Chaoyang, Qiqihar, Shanghai, Nanjing, and Jinan—Level II (low); Songyuan, Zunyi, Pu’er, Lhasa, Xining, and Yinchuan—Level I (very low).

Spatially, the high-risk areas were mainly located in East and Southwest China, whereas the areas with a medium-risk level were scattered throughout China (excluding Northeast China). In Central China, mostly medium-risk areas were found. The seven low-risk areas were mainly located in North, East, and Northeast China, and the six very-low-risk areas were distributed throughout high-elevation regions in the west, with a small number in the northeast.

**Figure 6:**
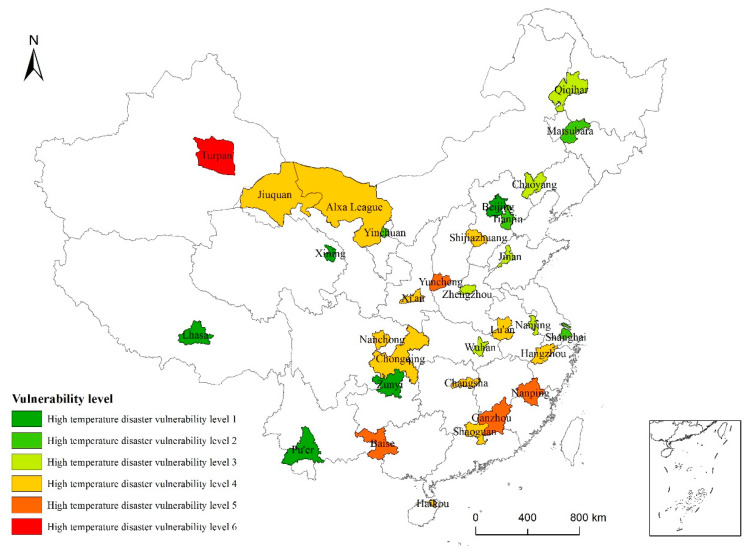
High-temperature vulnerability levels of 31 typical cities across China based on data from 1986 to 2015.

The highest vulnerability level was found for Turpan, which is consistent with the high-temperature risk for this city (Figure 6). However, there were significantly fewer very-high risk-level (Level 5) areas than high-temperature risk areas, and they were located in Southeast China (Nanping, Ganzhou) and western South China (Baise). Yuncheng, Chongqing, Hangzhou, and other cities with high-level risk values did not show high vulnerability levels. Areas with vulnerability levels of 3 and 5 were scattered across various regions, including Shijiazhuang and Alxa League in North China; Chaoyang and Qiqihar in Northeast China; Jinan, Nanjing, Hangzhou, and Lu’an in East China; Zhengzhou, Wuhan, and Changsha in Central China; Shaoguan and Haikou in South China; Nanchong and Chongqing in Southwest China; and Xi’an and Jiuquan in Northwest China. The Level 2 cities were Tianjin, Shanghai, Yinchuan, and Songyuan, and the Level 1 cities were Beijing, Zunyi, Lhasa, Xining, and Pu’er.

### 3.3. Comprehensive Assessment of High-Temperature Risk

**Figure 7:**
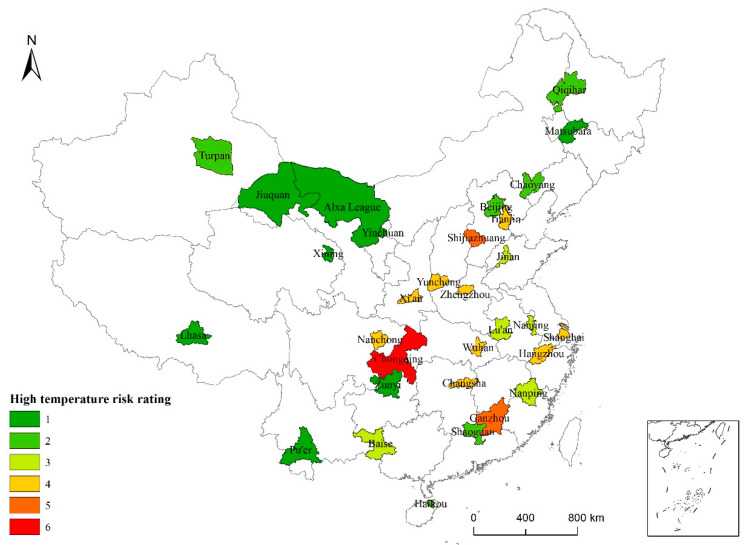
High-temperature risk distribution in 31 typical cities across China based on data from 1986 to 2015.

According to the high-temperature risk assessment model, the high-temperature risk values for the 31 cities were calculated and rated using the natural breakpoint method to obtain the spatial distribution map of high-temperature risk areas (Figure 7).

### 3.4. High-Temperature Risk Prevention Zoning

**Figure 8:**
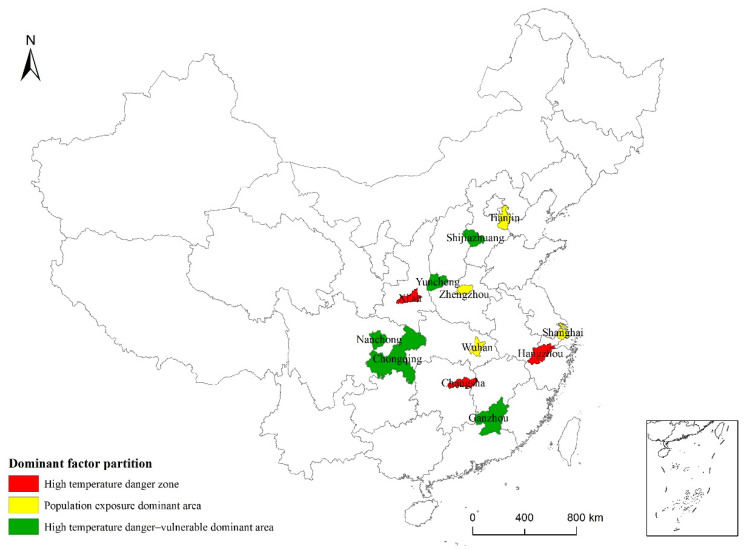
High-temperature risk dominant factor partition for 31 typical cities in China.

Risk prevention zoning is based on risk evaluation, which provides guidance for targeted risk prevention strategies [43]. In this paper, risk prevention and zoning were carried out for cities with higher high-temperature risk levels (Levels 4–6). First, the natural breakpoint method was used to divide the high-temperature risk and vulnerability factors into high and low levels. Subsequently, the cities with high risk and low vulnerability were called “high-temperature risk areas.” Cities with low risk and high vulnerability were called “high-temperature vulnerability areas.” Cities with both high risk and high vulnerability were called “high-temperature risk-vulnerability areas,” and cities with both low risk and low vulnerability were called “population exposure areas.” Among the 12 hotspot cities, there were 3, 0, 5, and 4 cities, respectively, in each of these four categories of risk factors (Figure 8); the highest risk values were found for Hangzhou, Xi’an, and Changsha. The cities with high risk and vulnerability values are Shijiazhuang, Yuncheng, Nanchong, Chongqing, and Guangzhou. In the densely populated cities of Tianjin, Zhengzhou, Shanghai, and Wuhan, there are no areas with high-temperature vulnerability. Based on risk prevention zoning, the areas subject to high-temperature risks are mainly located in plains, valleys, and river basins, whereas the areas with high vulnerability in terms of social aspects are mainly economically underdeveloped areas in densely populated regions.

The zoning of high-temperature cities based on the leading factors can be helpful in determining the mechanisms underlying high-temperature risks. Areas with significantly high summer temperatures can be identified by examining the natural environment, whereas areas with high vulnerability can be determined based on the distribution of vulnerable groups and physiological or socioeconomic conditions. Regarding population exposure, vulnerable groups can be spatially separated. Risk prevention strategies can be formulated according to different high-temperature risk factors. For areas with a high-temperature risk, increasing the amount of vegetation and green space can reduce temperatures; potential actions could be to increase vegetation density along the streets or to create green roofs. Urban planning departments should also consider methods to reduce high-temperature risks, such as the ventilation of new buildings, and residents should be made more aware of high-temperature risks. Regarding vulnerability, risk mitigation can be achieved by relocating vulnerable groups or by installing air-conditioning equipment. As a reduction in population density is not realistic, early warning systems should be considered.

## 4. Discussion

### 4.1. Analysis of High-Temperature Spatiotemporal Characteristics and Comprehensive Risk Assessment

This paper used meteorological and socioeconomic data to analyze high-temperature spatiotemporal characteristics and constructed a high-temperature risk assessment model. We comprehensively evaluated urban high-temperature risks by considering high-temperature characteristics, high-temperature risk, high-temperature vulnerability, population exposure, and risk-prevention zoning, expanding urban high-temperature risk assessment research. The spatiotemporal distribution characteristics and comprehensive risk assessment results were obtained for 31 typical Chinese cities, yielding different results when compared to Xie et al. [47] and Dong et al. [49], mainly for the following two reasons: (1) There are differences in the models; for example, Dong et al. [49] constructed their assessment model based on the aspects of disaster-causing risk and the vulnerability (exposure) of the supporting body, without considering social vulnerability. (2) There are also differences in the evaluation index systems; according to the different models, the selected evaluation index factors were different, but all models included factors such as the number of high-temperature days, population exposure, and economic development status.

The selection of relevant evaluation indicators is highly subjective. In the analysis of high urban temperatures, due to the limited amount of data, only the hottest cities in each province were selected for analysis, instead of evaluating all cities in China. The lack of data concerning the proportion of the population working in the primary industries of Lhasa, Jiuquan, and Turpan, as well as data concerning the local financial education expenditure, social security, and the employment expenditure in Pu’er, may have had a certain impact on evaluation results.

### 4.2. Spatial and Temporal Distribution Characteristics of High Temperatures in Typical Chinese Cities

The analysis of the high-temperature characteristics of Chinese cities based on interprovincial units is one of the key contributions of this paper. The spatial distribution characteristics of 31 typical cities in China were explained according to data from between 1986 and 2015. Over the past 30 years, the number of days with high temperatures gradually increased in nine cities, and the average number of high-temperature days in most cities increased by 5.44 days within 15 years. The increasing number of high-temperature days is the most obvious manifestation of climate warming.

### 4.3. High-Temperature Risk Assessment and Risk-Prevention Zoning in Typical Chinese Cities

A comprehensive risk assessment of high temperatures in Chinese cities, based on interprovincial units, is another key part of this paper. On the basis of the natural disaster risk evaluation framework, a high-temperature risk assessment framework based on “high temperature risk–social vulnerability–population exposure” was constructed. Quantitative analysis was carried out based on the spatiotemporal characteristics of high temperatures, high-temperature risk, and high-temperature vulnerability. Finally, comprehensive evaluations and risk-prevention zoning were carried out for urban areas with high temperatures according to the following three aspects: the duration of high-temperature events, the severity of high-temperature events, and the extreme high-temperature risk based on weather characteristics. Vulnerability was estimated using the high-temperature risk and social vulnerability. Finally, by combining the high-temperature risk, high-temperature vulnerability, and population exposure, the high-temperature risk levels of the 31 typical cities were obtained. Generally, cities with high risk levels also showed high vulnerability levels; this was true for cities such as Turpan, Nanping, Ganzhou, and Baise.

Generally, areas with high risk levels, social vulnerability, and large populations being exposed to high temperatures are located in plains, valleys, and river basins; they are largely economically underdeveloped and densely populated areas. For high-temperature areas, increasing the vegetation density and creating green rooftops can be effective measures. Risk mitigation can be achieved by resettling vulnerable people. Regarding population exposure, early warning systems and evacuation strategies should be taken into consideration. These results can provide a realistic basis for decision making for meteorological departments and disaster prevention and mitigation departments; they have a certain guiding significance for understanding the regional high-temperature disaster risk and the vulnerability of disaster-bearing bodies, and they contribute to regional high-temperature risk management, high-temperature risk avoidance, and risk control.

## 5. Conclusions

Due to global climate change, extremely hot weather conditions are becoming frequent. Based on the provincial unit, the characteristics of high temperature in Chinese cities are analyzed. The results show that over the past 30 years, most cities have experienced 5–8 significant oscillation periods in terms of the number of high-temperature days, and the number of high-temperature days in nine cities, including Tianjin, Shanghai, and Chongqing, shows a significant positive time correlation. A comparative analysis of two 15-year intervals shows that in 87% of cities, from 2001 to 2015, the average number of high-temperature days per year increased by 5.44 days compared with 1986–2000.

By conducting a comprehensive assessment of the high-temperature risks, it was observed that the areas with the greatest high-temperature disaster risk in China are mainly concentrated in the central urban areas of plains, basins, and river basins. These areas have low social vulnerability due to the development of cities, but, similarly, the urban high-temperature risk and the exposure of the population to high temperatures are much higher than they are for cities in the western regions; thus, these cities have a significant high-temperature disaster risk.

The results of risk-factor zoning show that the areas with the greatest high-temperature risk are mainly plains, basins, and river basins, and the areas with the highest social vulnerability mainly include economically underdeveloped areas and areas where socially vulnerable people gather. The areas where a large proportion of the population is exposed to high temperatures consist mainly of densely populated areas.

## Figures and Tables

**Table 1:** Description of the data used for this paper.

Data Types	Data Description	Data Sources	Time Period
Meteorological data	Daily maximum temperatures from 194 meteorological stations	National Meteorological Science Data Sharing Center (http://data.cma.cn/site/index.html, accessed on 15 Apirl 2020)	1986–2015
Socio-economic data	Statistical data, including population, employment, income, finance, industry, education, healthcare, and other data from various administrative areas	Provincial statistical yearbooks from Anhui, Gansu, Guangdong, Guangxi, Hebei, Henan, Heilongjiang, Hubei, Hunan, Jilin, Jiangxi, Liaoning, Inner Mongolia, Shandong, Shanxi, Sichuan, Tianjin, Tibet, Xinjiang, Yunnan, Chongqing, Shanghai, Hainan, Beijing, Zhejiang, Guizhou, Qinghai, and Ningxia. Municipal statistical yearbooks from Nanjing, Nanping, Wuhan, Chaoyang, Shijiazhuang, Xi’an, Yuncheng, Kunming, and Zunyi. Statistical yearbooks are all from provincial and municipal statistical bureaus.	2016
	Statistics (As supplementary materials)	Heilongjiang Financial Yearbook and the national, economic, and social development statistical bulletins from the cities of Ganzhou and Pu ’er and other provinces and cities, provided by the Provincial and Municipal Statistics Bureau	2015

**Table 3:** Time series analysis of high-temperature days from 1986 to 2015.

Statistical Metric	Tianjin	Shanghai	Ganzhou	Zhengzhou	Changsha	Nanchong	Chongqing	Yinchuan	Turpan
Pearson correlation	0.368 *	0.460 *	0.488 **	0.443 *	0.526 **	0.424 *	0.465 **	0.679 **	0.614 **
Sig. (2-tailed)	0.045	0.011	0.006	0.014	0.003	0.020	0.010	0.000	0.000
N	30	30	30	30	29	30	30	30	30

* Significant correlation at the 0.05 level (bilateral). ** Significant correlation at the 0.01 level (bilateral).

**Table 2:** Indicators used for the social vulnerability index calculation.

Primary Indicator	Sub-Indicator
Sensitivity	Proportion of the population that is female (%)
	Proportion of the population that works in the primary industry (%)
	Registered unemployment rate (%)
	Number of students in primary school (people)
Adaptability	Per capita disposable income of urban residents (CNY)
	Per capita disposable income of rural residents (CNY)
	Basic endowment insurance for urban workers (CNY)
	GDP per capita (CNY)
	Proportion of industrial output value in GDP (%)
	Local fiscal revenue (CNY 10,000)
	Number of health technicians (people)
	Local financial education expenditure (CNY 10,000)
	Social security and employment expenditure (CNY 10,000)

## Data Availability

All relevant datasets in this study are described in the manuscript.

## Appendix A. PCA Analysis and SVI Calculation

We used the SPSS software platform to perform a principal component analysis of the standardized indicators and to calculate the social vulnerability of each city. The original index must pass the KMO test, which is a prerequisite of using the principal component analysis method. According to the standard provided by the statistician Kaiser, a KMO value of less than 0.6 means that the data are not suitable for factor analysis. KMO = 0.706 in this paper, so the data meet the requirements of principal component analysis. The orthogonal rotation method with maximum variance was used to make the coefficients in the factor load matrix more significant, and the initial factor load matrix can be rotated so that the relationships between the factors and the original variables can be redistributed and the correlation coefficient can be restricted to a range from 0 to 1.

The principal component analysis of the standardized data was carried out using the SPSS software platform, and four principal components—the characteristic values of each principal component, the contribution rate of each principal component, and the score coefficient matrix of each component—were obtained (Table A1 and Table A2). Four principal components were obtained from the 13 variables in the social vulnerability index system, and the cumulative contribution rate was 83.66%.

**Table A1:** Time series analysis of high-temperature days.

Eigenvalue	Contribution Rate	Cumulative Contribution Rate
4.875	37.500	37.500
3.474	26.724	64.224
1.270	9.769	73.993
1.257	9.671	83.664

**Table A2:** Component function matrix.

Components of Component Function Matrix				
X	Z1	Z2	Z3	Z4
X11	0.015	−0.073	0.623	−0.208
X12	−0.140	0.313	−0.146	0.049
X13	−0.083	−0.008	0.017	0.767
X14	0.199	0.010	−0.066	−0.107
X15	0.002	0.240	−0.040	−0.028
X16	−0.045	0.283	0.008	−0.067
X21	−0.109	0.302	0.200	0.043
X22	−0.076	0.068	0.576	0.262
X23	0.198	0.038	−0.056	−0.231
X24	0.188	−0.130	0.109	0.108
X25	0.212	−0.015	−0.014	−0.112
X26	−0.210	0.203	0.037	−0.286
X27	0.229	−0.066	−0.034	−0.045

To calculate social vulnerability, we first calculated the principal component score function of each typical city according to the obtained component score coefficient matrix:

(A1) $$F_{i}=\sum^{\;}Z_{ij}\times X$$

where *X* represents the standardized numerical value of each index, and *Z_ij_* represents the corresponding component score of the index. Then, the social vulnerability value of each typical city was calculated using the contribution rate of each principal component as follows.

(A2) $$F=\frac{37.5}{83.664}F_{1}+\frac{26.724}{83.664}F_{1}+\frac{9.769}{83.664}F_{1}+\frac{9.671}{83.664}F_{1}$$

Here, *F* represents social vulnerability, and *F*_1_, *F*_2_, *F*_3_, and *F*_4_ represent the scores of each principal component, respectively.
